# A gravity model for emergency departments

**DOI:** 10.1038/s41598-025-99840-w

**Published:** 2025-06-04

**Authors:** Francesco Bertolotti, Fabrizio Schettini, Federica Asperti, Emanuela Foglia

**Affiliations:** 1 School of Industrial Engineering, LIUC - Carlo Cattaneo University, Corso Matteotti, 22, 21053 Castellanza, VA Italy; 2 Healthcare Data Science Lab (HD-LAB), LIUC - Carlo Cattaneo University, Corso Matteotti, 22, 21053 Castellanza, VA Italy

**Keywords:** Gravity model, Emergency departments, Facility location, Healthcare, Health policy, Health care economics, Computer science, Applied mathematics

## Abstract

The issue of facility location is of significant importance in numerous systems, where the efficient utilisation of resources is of great importance. Gravity models, which are inspired by Newtonian physics, are commonly employed to address these problems and have a long tradition of being used in healthcare. The objective of this paper is to enhance the comprehension of patients’ decision-making processes in emergency healthcare by introducing an extension to existing gravity models, which includes two novel factors influencing emergency department choice: hospital sizes and patients’ severity. The newly formulated gravity rule, which integrates these factors, demonstrated an extremely high accuracy against real-world data in terms of overall hospital location and flows between cities and hospitals, respectively 98.77% and 98.02%.

## Introduction

The facility location problems are relevant across various systems due to their critical role in the efficient utilization of resources^1^. Usually, these problems can be solved by determining the better locations for a set of facilities in a given solution space, in such a way that maximizes users’ accessibility and minimizes operational costs, thereby ensuring effective resource allocation^2^. This decision-making process is crucial in different sectors such as logistics^3^, urban planning^4^, healthcare^5,6^, and, in general, in every context where the facility placement affects the transportation costs, the level of services, and the operational effectiveness^7^.

Various methodologies have been developed to address location problems, each offering specific advantages. For example, linear programming is widely employed for solving location problems due to its effectiveness in handling large-scale linear models^8^. However, it cannot be always employed when the problem structure does not permit the representation of real-world features with linear functions^8^. Metaheuristic optimization presents a more flexible alternative^9^ since this method is not confined to linear constraints and can be efficiently employed when the solution spaces are complex^10^, at the cost of a much higher computational effort^11^. Also, qualitative methodologies such as Delphi methods have been employed^1^.

A noteworthy specific case of the facility location problem arises when one or more locations should serve a large number of users distributed in a given space^12,13^. To effectively undertake such challenges, a possible approach involves leveraging the assumption that the potential customers’ likelihood of visiting a facility is inversely proportional to their distance from it^14^, mirroring the Newton’s law of universal attraction. The models that employ this metaphor are aptly termed ’gravity models’^15^, and can include other non-physics related features^16^.

Gravity models have been employed to deal with different problems, such as the interpretation of the trade flow between two nations using GDPs as function of inter-country distances^17^, the location of new logistics hubs^18^, and the inference of retail facility attractiveness from secondary data regarding customers’ buying power and sales volumes^19^. In healthcare, gravity models have also been utilized for many years^20,21^, offering insights for the decision-making process and policy formulation, with interesting potentialities for hospitals’ improvement and application in various healthcare services’ settings^22–26^.

This paper endeavours to enhance the current understanding of the patients’ decision-making process in emergency healthcare scenarios, improving the state-of-the-art related to gravity models^16,25,27,28^, Table 1 summarizes the main contributions already existing in literature. The contribution to the field regards the way two new factors, in addition to distance, influence the choice of emergency care department^29^: the hospital size and the patients’ perceived severity. The model relies on the assumption that a larger hospital, presumably with more extensive facilities and resources, is more likely to be the preferred choice for patients^30^, and that this preference is amplified by the patients’ perceived severity of their condition. Especially, the more critical the condition, the greater the likelihood of a patient opting for a larger hospital. To the best of our knowledge, this hypothesis has never been investigated before, but it could be extremely relevant to better understand patients’ behaviour and improve emergency departments facility locations. The novel gravity rule underwent a calibration and validation phase on real-world data from emergency care units from Lombardy (Italy)^31^, to gain relevancy to policy-makers^32,33^, which yielded results that surpassed current benchmarks in the field. The outcome of this empirical testing shows that the mean error between real-world data and the results simulated with the gravity model is approximately $1.23\%$.

## Research objects and methods

This section presents the proposed gravity model and its calibration to a specific geographical area, firstly providing a formal description of our gravity meta-model for hospital selection, detailing the foundational modeling hypotheses, the variables incorporated into the model, and their interplay; then the application of this model in a specific context is illustrated including a thorough explanation of the methodologies and procedures employed in our experiments, ensuring scalability and generalizability of our findings. The implementation of the model has been performed in Python 3.11.3. The notebook utilized for data analysis, along with a representative subset of the data used and the specific packages and versions employed in the analysis, are publicly available.

### Gravity meta-model

This research focuses on developing a function that accurately model the patient likelihood of choosing a particular hospital, considering distance and various other determinants. Unlike existing models that often rely on general preference data, this approach is event-based specifically targets the final stage of the decision-making process^34^. This stage captures the definitive choice a patient makes, either independently or through their transportation medium, in cases where the patient is not autonomous. Thus, the structure of the candidate class of functions is as follows:

1 $$\begin{aligned} p_k^*(H_i) = f(d_{k,i},t,b_i) \end{aligned}$$

where $p_k^*(H_i)$ stands for the supposed preference of a patient *k* to choose the hospital $H_i$ among all the *N* hospitals available. $d_i$ is the distance between patient *k* and the hospital *i*, $t_k$ the triage code upon arrival, which represents the severity of patient *k*, and $b_i$ the average perceived size of hospital *i*, serving as a proxy for its emergency care department accessibility, which is the same for every patient *k*.

The distance $d_{k,i}$ is computed using the public API from the Open Source Routing Machine using Python 3.9 on the 13th September, 2022. The metric was selected under the hypothesis that the preference towards a specific hospital is affected by the duration of the travel and not by the actual routing distance, given that in Italy no triage tool or guideline dictates which hospital a patient should choose when seeking emergency care. Thus, the traveling time between every city in the area of analysis was collected, measured in seconds. Also, since it is not possible to gain precise information regarding the exact place where a patient *k* starts, it was hypothesised that, for area inhabitants, the residence town is a sufficiently good approximation of the town where they are located when they decided to which hospital to go^35^.

The triage code $t_k$ is assigned to each patient *k* at the entrance of an emergency care department and embodies the evaluation of patients’ severity made by nurses when the patient arrives at the emergency department of reference. In the considered period, the Italian healthcare system categorized the severity of a patient’s condition using color codes, in ascending order of severity: white, green, yellow, and red, which are respectively encoded in a non-ordinal numerical variable whose value can be 1, 2, 3, and 4. A further code, the black one, also exists to include patients that are deceased upon arrival at the emergency department. This model considers the triage code as a proxy for the seriousness of the condition of a patient at the moment they leave their residence, not including the black codes in the analysis. This classification was later updated, but at the time of data collection a four levels scale was adopted. The triage code is determined upon the patient’s arrival at the emergency department, following an initial medical evaluation conducted by healthcare professionals. Consequently, triage does not play a role in the patient’s initial decision regarding which hospital to visit.

The parameter $b_i$ stands for the size perceived by a patient regarding a specific hospital $H_i$ during the decision, given that data regarding individual preferences are not available. The rationale is that a hospital with more beds could be considered to have a “higher” quality than a hospital with fewer beds^36^. The number of beds was employed as a proxy for hospital size, as it is easily accessible and reflects both patient capacity and medical specialization breadth. Larger hospitals can treat a wider range of conditions, including severe cases, and have greater capacity to admit patients without transfers. From a patient perspective, this can then relates to the perceived quality. We use the term “perceived” because patients, in a situation of bounded rationality, are not supposed to know the exact services provided by each hospital; even so, they do not have the time and the resources to process this information. Also, even if a survey were conducted, the results could be affected by two elements. First, the answer would not be given in a moment of stress, such as the one in which a patient is choosing which emergency care department to direct to. Second, it would not include the decision-making of rescue vehicle drivers, which should be obtained by means of a second survey. Since the goal of this work is to find a universal and simple rule that can simulate the distribution of individuals in different areas, we used as a proxy of the perceived accessibility parameter the number of beds in the emergency care department of the hospital $H_i$. This metric takes into consideration both the perception of private individuals and rescue vehicle drivers, since all of them are affected in their location selection process by the size of the hospital, and it has also high adherence to reality, since the dimension of the hospital is likely connected to the number of healthcare services that it is able to offer, representing a direct measure of the accessibility to the healthcare system^37^.

Given these hypotheses, the following preference function is proposed.

2 $$\begin{aligned} p_k^*(H_i) = \frac{b_i^{\alpha t_k}}{d_{k,i}^\beta } \end{aligned}$$

The function combines $b_i$, *t*, and $d_i$ by raising the perceived accessibility to the power of *t* and dividing it by the distance. $\alpha$ and $\beta$ regulate the effect of each factor. This equation implies that as the perceived accessibility increases or the severity of the patient’s condition intensifies (higher $b_i$ and *t* values), the preference for a particular hospital $p^*(H_i)$ also increases. Conversely, there is an inverse relationship between the distance from a hospital $d_{k,i}$ and the preference toward it, as that the longer it takes to get to the hospital, the less likely it is for a patient to direct there.

From all the *N* preferences $p^*(H_i)$, a probability for a patient to head to the $H_i$ structure can be derived as

3 $$\begin{aligned} p_k(H_i) = \frac{p_k^*(H_i)}{\sum _{i=1}^{N} p_k^*(H_i)} \end{aligned}$$

Maintaining the metaphor of gravity, the probability for a patient to go to the hospital $H_i$ in a given condition is proportional to the attractiveness of $H_i$.

**Fig. 1:**
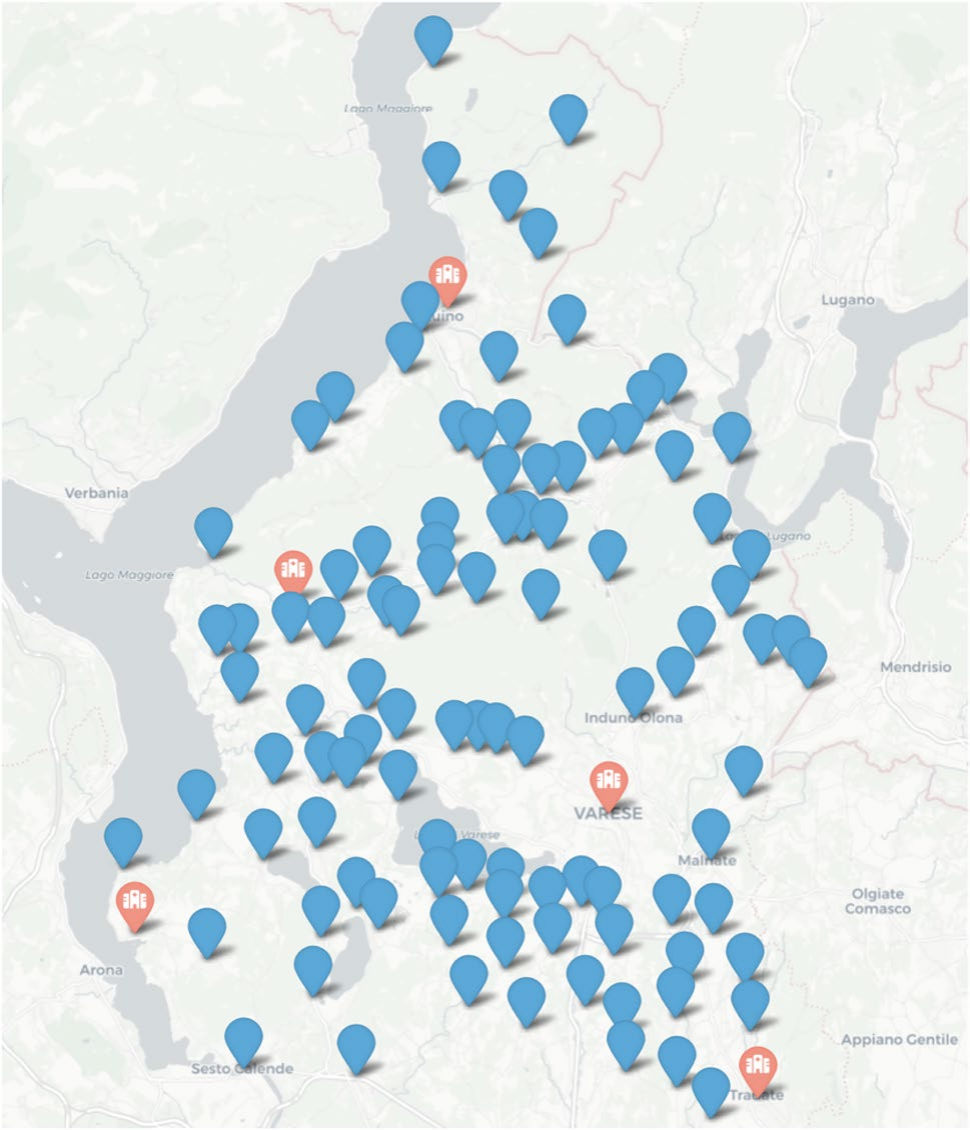
Geographical position of the towns in the considered area, divided for town where no emergency departments are present (red marker) or absent (blue marker).

### Model specification

The meta-model presented in the previous paragraph was adapted to a specific area, the region served by the hospitals associated with the Sette Laghi Territorial Social and Healthcare Organizations (ASST), a healthcare facility located in the northern part of Lombardy in Italy. Figure 1 depicts the geographical distribution of cities in the area and the cities where an emergency care department is present. Specifically, this study focuses on six hospital facilities, each with a corresponding emergency department, which provides comprehensive medical aids to the population, including specialized treatments, diagnostic services, and emergency care. The ASST includes six different emergency departments, summarized in Table 2, with the number of beds that have been used to calibrate the model. One could argue that the patients’ perception regarding a hospital do not depend only on the size of the emergency care department, but also from the overall number of beds of the whole hospital; so, Table 2 also reports the total number of beds. Since they strongly linearly correlates, the number of beds in the emergency care department was employed in the calibration and validation, as it was more in scope with the purpose of the model.

The dataset utilized in this study comprises patient behavior records in the specified region, encompassing patient arrivals at emergency departments during 2019, the second half of 2021, and the first half of 2022. This dataset documents a total of 325,886 arrivals. Each entry details relevant information, such as the severity level and the patients’ city of residence. For the purpose of this analysis, patients not residing in towns within the ASST Sette Laghi area were excluded, resulting in a consideration of 256,701 emergency department arrivals.

The process of model calibration involves determining the optimal values of parameters $\alpha$ and $\beta$ to minimize the discrepancy between the observed and simulated distributions of resident arrivals at emergency departments within the study area. The simulation of the distribution is executed as follows: a random subset of 25,000 records, each representing a patient arrival at an emergency unit, is extracted from the dataset. For each record, a selection probability $p(H_i)$ is calculated for each hospital $H_i$. Subsequently, a destination hospital $H_i^*$ is chosen based on this probability distribution, and the patient is assigned to that hospital. Upon allocating all 25,000 patients, a specific metric is utilized to evaluate the divergence between the actual and simulated distributions for each hospital.

4 $$\begin{aligned} p_k^*(H_i) = f(d_{k,i},t,b_i) \end{aligned}$$

where ${f_i}^r$ and ${f_i}^s$ respectively the relative frequency of $H_i$ for real data and simulated data. A genetic algorithm is then employed to estimate $\alpha$ and $\beta$ minimizing $max(e_i)$. The algorithm runs for 250 generations, with a population of 5,000 possible solutions.

## Results

The results divide into three distinct parts. The first encompasses an aggregate analysis, which underscores the efficacy of the model in accurately allocating the population to the appropriate emergency care departments, while the second focuses on how this allocation is achieved, emphasizing the retention of the unique characteristics inherent in the allocation network. Finally, the patient allocation difference between real-world data and simulated data is shown for each city. This threefold approach was adopted to provide the most comprehensive understanding of the proposed methodology performance and its precision with a progressively higher level of detail.

**Fig. 2:**
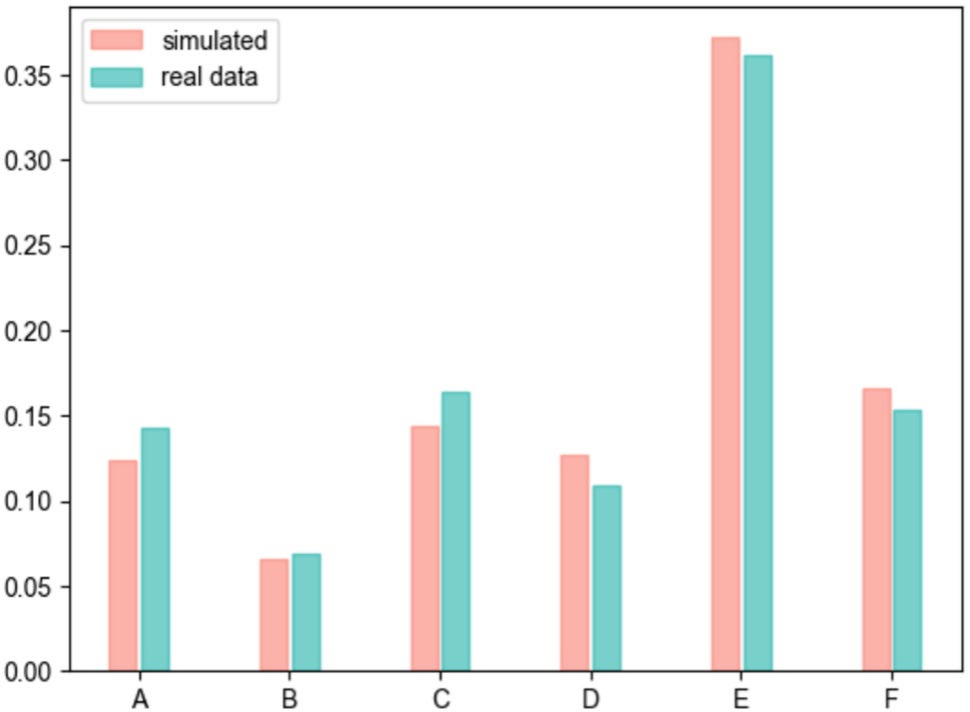
Comparative analysis of the relative distributions of patient arrivals at each hospital level.

**Fig. 3:**
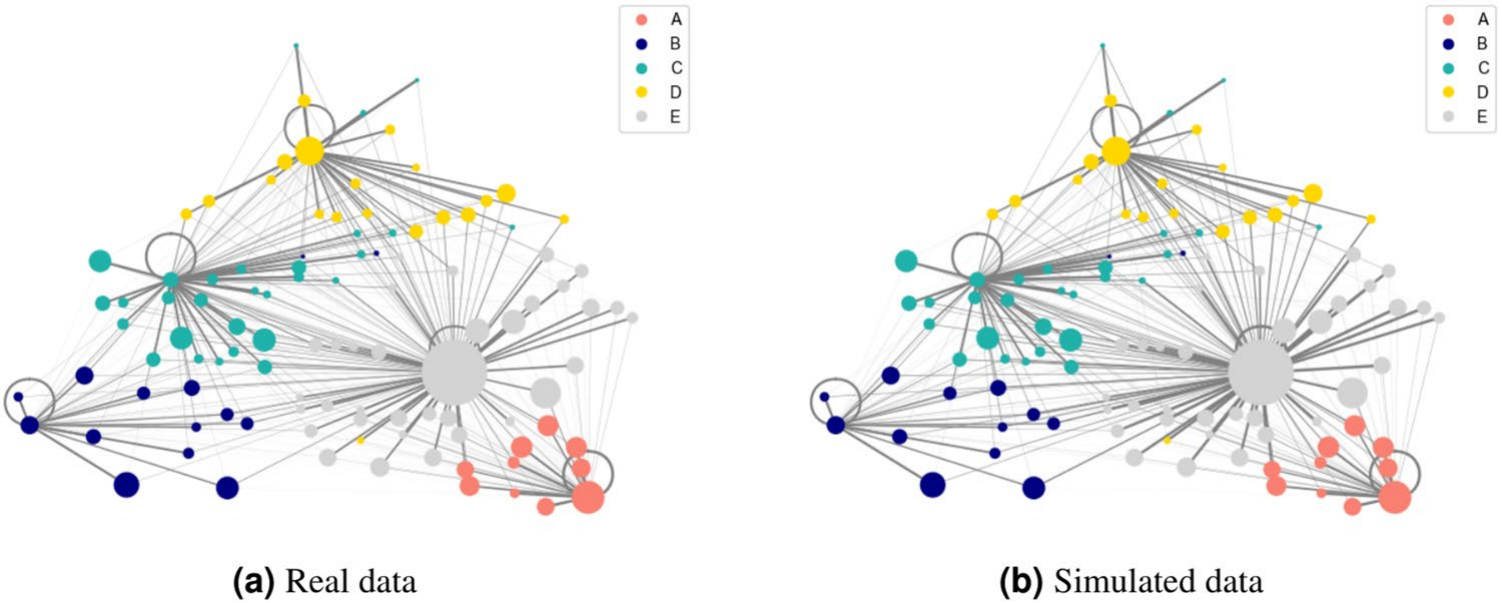
Comparison of the network of distributions between real data and the results of the simulated data. The color stands from the targeted city in which most of the population has gone, while the size embody the population of the corresponding city.

**Fig. 4:**
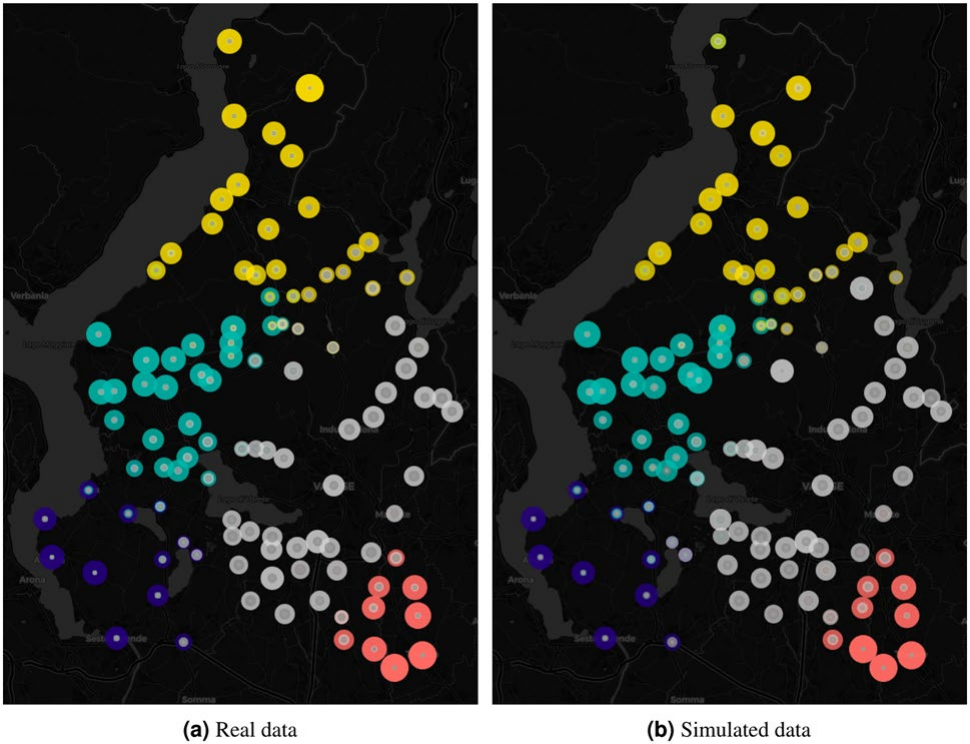
Share of the patients heading to a given hospital per each city.

**Table 1:** List comprises the primary references that utilize gravity models in the context of healthcare systems.

Source	Nation	Organization	Objective	Parameters	Accuracy
Mayhew (1984)	UK	Hospitals	Review of gravity models applied to healthcare	Demand, treatment resources, accessibility cost	n.a.
Congdon (2000)	UK	Emergency Departments	Predict hospitalization flows	Demand, distance from patient’s home, distance-based accessibility	n.a.
Congdon^21^	UK	Emergency Departments	Predict hospitalization flows	Demand, distance from patient’s home, distance-based accessibility	n.a.
Lowe and Sen^20^	USA	Hospitals	Forecast changes in patient flow	Hospital success, travel time, # trips from home to hospital, patient insurance status	n.a.
Congdon (2010)	UK	Emergency Departments	Estimate and predict hospitalization flows	Demand, distance from patient’s home to hospital, distance-based accessibility	n.a.
Teow et al. (2017)	Singapore	Hospitals	Estimate demand for new hospitals	Patient days at existing hospitals, patients’ residence, hospitals’ gravity mass coefficient	76%
Latruwe et al. (2022)	Belgium	Hospitals	Optimize facility location and hospital admissions	distance (great-circle distance, travel time by car, and distance-time), hospital size, transport accessibility	85–90%
Shi et al. (2022)	China	Hospital	Assess the spatial accessibility patterns	Hospitals’ capacity, service ability, distance from patient’s home, population distribution, and transport accessibility	n.a.

**Table 2:** Hospitals with emergency department in the ASST Sette Laghi, with the number of beds places in the hospital.

Hospital code	City	# Total beds	# beds in ED
1	A	112	25
2	B	40	10
3	C	86	15
4	D	44	13
5	E	469	84
6	E	139	15

Figure 2 displays the relative frequency of allocating the population from a city to a hospital, as delineated in the model specification section. The experimental results indicate that the maximum discrepancy between any two elements in the relative distribution is $max(e_i) = 0.0215$, while the average error is quantified at $E[e_i] = 0.0123$. This implies that the calibrated model can assign each patient, based on their triage code and city of residence, to the appropriate hospital with an average precision of 0.9877.

Figure 3 presents a network visualization of this complex system^38,39^, where each node represents a city, positioned according to its geographical coordinates (longitude and latitude). Each link denotes a movement of inhabitants from the residency city *x* to an emergency department in another city *y* where an hospital $H_i$ is located. The color of each node indicates the city of the emergency department to which the majority of a city’s population travels, while the node size corresponds to the population size of the city. The comparative analysis of these networks provides two observations. Firstly, employing travel time as a metric, as opposed to Euclidean distance or travel distance, preserves geographical proximity, confirming the proposed model’s adherence to real-world spatial relationships. Secondly, it is evident how the model not only accurately allocates individuals to hospitals but also precisely reconstructs the network of connections between cities of residence and hospital locations^40^. This dual capability of the model highlights its effectiveness in both individual allocation and in mapping the broader network of healthcare access.

To enhance the reliability of the results and to add a quantitative perspective to Fig. 3, a final analysis was conducted to assess the precision of the model’s allocation of each city’s population to a hospital. This analysis evaluates the proportion of individuals from a residency city *x* attending a hospital *i* in both real data and the outcomes predicted by the gravity model. Figure 4 shows the comparison of the proportion of patients traveling from a specific town to a given hospital. The sub-figures distinguish between real and simulated data, while the color code is consistent with that used in Fig. 3. The graphs suggest that, with some minor variation, the share of patients traveling from a residency city *x* to a hospital *i* can be accurately reproduced by the model.

**Fig. 5:**
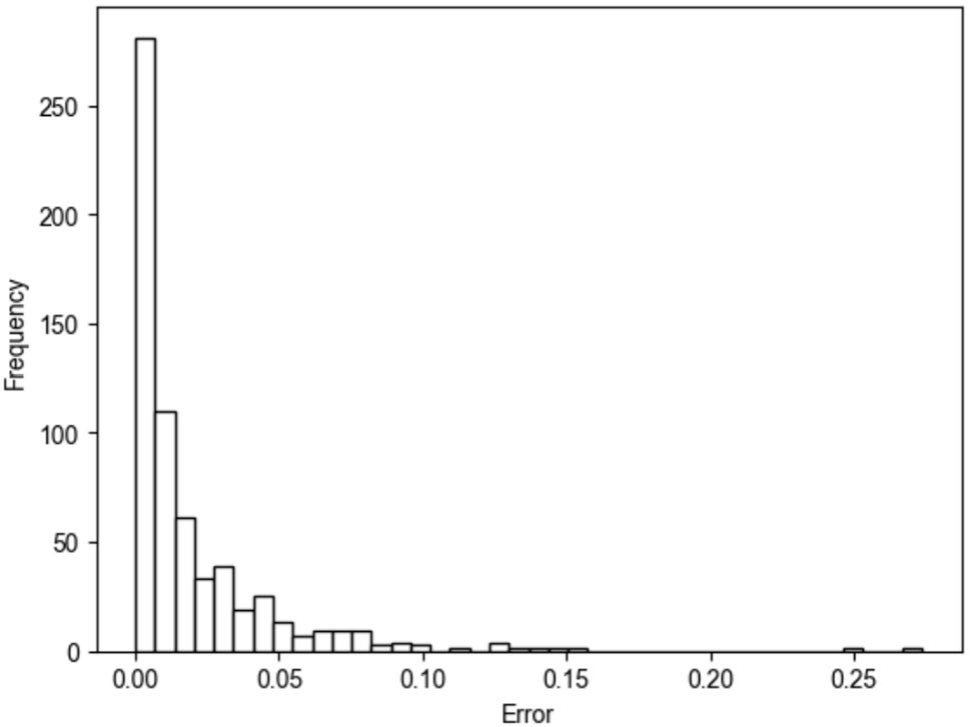
Distribution of the error of evaluation for each combination of city of origin an city of the hospital.

Finally, we quantify the allocation error for each residency city-destination hospital pair as $e_{x,i}$, the distribution of which is illustrated in Fig. 5. Although the distribution is right-skewed, indicating some higher values, the average error across all cities is $1.982\%$, and the median error is $0.897\%$. Nevertheless, the majority of the errors come from small towns. For towns with a population above 7,000 inhabitants, which is the threshold for city classification in Italy, the mean error is only $0.699\%$.

## Discussion

### Results discussion

This results from the study presents several significant implications, both in terms of theoretical advancements and practical applications.

From an academic perspective, it represents a further step in understanding patient behavior when seeking emergency care. By incorporating hospital size and patient severity into the gravity model framework, this work extends previous models and provides a refined approach that could be adapted to other domains with similar features.

For policymakers, these findings offer valuable insights that can directly influence healthcare infrastructure planning. The model can be employed to guide the optimal placement of new emergency care facilities, ensuring a balance between system efficiency and patient accessibility, minimizing patient travel distances and waiting times and ultimately improving the overall service level. Additionally, the insights derived from this study can support resource allocation strategies, helping to redistribute medical personnel across facilities and consequently mitigate emergency department overcrowding.

Finally, this paper has also direct clinical implications. The model’s predictions could inform strategies for inter-hospital patient transfers, ensuring that critically ill patients are directed to facilities best equipped to handle their conditions. Furthermore, communicating these findings to the general public could enhance awareness of hospital selection processes, encouraging more informed and rational decision-making, especially in cases where patient choices are influenced by misperceptions rather than objective factors.

Morover, the gravity model introduced in this paper demonstrates precision in two key aspects: the specific allocation of individuals to hospitals, which is executed with high accuracy (as illustrated in Fig. 2), and in determining the contribution of cities, denoted as *j*, to specific hospitals, labelled as *i*, in terms of patient flow (see Figs. 3 and 5).

Common sense and previous studies suggest that the workload of a hospital is influenced by the population size and the availability of alternative healthcare options in the vicinity^20,28^. This gravity model provides a means to account for these factors, suggesting how factors different than physical proximity, such as the attractiveness of an hospital, could help to better fit the data^24^. Usually, these fine-tuning operations usually complicate the model, and even when they have a great fit with data, they become less explainable with every new assumption^41^. The main merit of this model is to explain a complex behaviour with a very simple rule^42^, employing simplifications such as the absence of personal networks^43^.

Drawing from the previous outcomes, discernible spatial patterns become evident concerning the possible geographical accessibility to emergency care departments within the specified territories examined in the analysis, when the conditions of patients are taken into account. One significant inquiry arises: what are the underlying reasons for the distinctive spatial pattern observed in terms of potential access to emergency departments?^22^ While factors such as topography and historical settlement patterns have certainly influenced the current distribution of the population^34^, it is evident that these spatial patterns cannot be solely attributed to them. Another crucial explanatory factor is the decision-making process in healthcare services, which has decided both the position of the emergency department and the assignation of a fourfold progressive code to classify the severity of a patient’s condition. These systemic factors are likely to contribute to the spatial patterns identified through the modified gravity model. Nevertheless, their precise role remains uncertain at present. To address this knowledge gap, it would be necessary to engage in consultations with decision-makers responsible for resource allocation, system design, and other key functions^22^.

Finally, while the analysis was conducted on a region with a single major urban center and its surrounding areas, the model’s underlying principles remain valid across different urban settings.The model proposed in this paper abstracts the territory and the healthcare issue. Therefore, the fundamental mechanisms governing hospital accessibility and patient distribution are not affected by territorial distribution: greater travel distances would still reduce the likelihood of a patient reaching a specific hospital, while larger hospitals still would be more likely to receive critically ill patients due to their capacity and specialized services. However, in the context of larger metropolitan areas, where multiple hospitals may coexist within a densely populated region, a finer granularity in data could be necessary. Unlike the case considered in this study, where not city required subdivision, urban environments may demand a more detailed representation of hospital catchment areas, partitioning them into multiple nodes. A crucial factor in adapting the model to urban settings is the availability of patient location data. If hospital information systems provide anonymized address data for patients arriving at emergency departments, it becomes feasible to refine the model by mapping patient flows with higher spatial precision.

### Limitations

Even if yielding promising results, this study is subject to certain limitations that merit consideration. Firstly, there is the need for further validation in diverse geographical areas, to validate the model with more diverse data^44^. Designed with generality in mind, the model is theoretically applicable to any region where Geographic Information System data are available and patient arrivals at the emergency department are encoded into a hierarchical classification system. However, the current research does not assert that the relationships identified are universally applicable across different contexts or areas, and further empirical studies are needed to assess the model’s adaptability and efficacy in other settings.

Another notable limitation pertains to the distinction between patients’ residential locations and the actual departure point. The present model solely accounts for the former, lacking data on the latter. This absence of information about the actual starting location of emergency care journeys may limit the accuracy of the model, even thought the results are very promising. The gravity model demonstrates robust results when analyzing patient allocation based on residential areas, but a more precise fit to the data might remain impracticable without the integration of additional information. It highlights the need for an even more comprehensive data collection.

Notably, in the considered setting, there are no on-site triage tools or guidelines to determine which hospital a patient should attend when emergency care is required. On the one hand, this can be considered a positive aspect, as patients’ decisions are not externally directed, and we could observe their effects. This allows for the investigation of their decision-making processes, as the effects can be observed directly in the data. On the other hand, the absence of standardized criteria leaves the evaluation to the initial medical assessment conducted by healthcare professionals in the emergency care units, thereby introducing a degree of subjectivity.

A limitation of the present approach concerns the use of the parameter $b_i$ as a proxy for the hospital size as perceived by patients during the decision-making process, in the absence of data on individual preferences. The number of hospital beds was adopted as an indicator of perceived quality, based on the assumption that larger hospitals are more likely to be associated with higher capability and comprehensiveness of care^36^, but not specific information regarding the belief of patients regarding each hospital $H_i$ has been gathered. Furthermore, the actual capacity of a hospital is influenced by more than just the number of beds; other elements, such as staff and materials, can also impact the quality and quantity of care that a given structure can provide.

### Future developments

Several avenues for future research have been identified to further substantiate and expand upon the current results.

First, replicating the current analysis across a broader and more diverse range of territories could validate the results obtained in this study and test the applicability and robustness of this gravity model extension in different geographic and demographic contexts, increasing the scientific relevance of the work^45^. Also, further analysis could assess whether the patterns and relationships identified are consistent across various settings or depends on specific geographical features^46^.

Second, developing a simulation model could additionally confirm the results of this study^47^. For example, an agent-based model simulating the interactions of individual patients within a healthcare system could offer a powerful tool for testing hypotheses and observing emergent behaviours under varying conditions^48^.

Finally, causal inference model of micro individual behaviour aimed at directly confirming the relationship between individual decision-making processes and the variables identified in this study as influential factors in patient behaviour^49^ might be designed.

## Conclusion

This study presents a novel gravity-based model for emergency department selection that integrates traveling distance, hospital size, and patient severity. By incorporating these variables into a unified framework, the model achieves to remarkably simulate real-world patient flows. Its simplicity, interpretability, and empirical validation make it a valuable tool for both researchers and decision-makers. The model highlights how perceived accessibility and severity-driven decisions can shape healthcare dynamics beyond mere geographic proximity. Although developed in a specific regional context, the underlying principles are broadly generalizable.

## Data Availability

The code and a subsection of the data used for the experiments are available in the following repository: https://anonymous.4open.science/r/gravitymodel-submission. The complete data are available upon reasonable request, which should be made to fbertolotti@liuc.it.
